# Social Media Usage Patterns and Anxiety in Chinese Adolescents: Age-Specific Risks from a Cross-Sectional Perspective

**DOI:** 10.3390/bs16101895

**Published:** 2026-10-14

**Authors:** Juan Liu, Ruizhi He, Bijie Tie, Xueyang Wang, Jiang Qiu

**Affiliations:** 1 Key Laboratory of Cognition and Personality (SWU), Ministry of Education, Chongqing 400715, China; lj__edu@163.com (J.L.);; 2 Faculty of Psychology, Southwest University, Chongqing 400715, China; 3 School of Psychology, Shaanxi Normal University, Xi’an 710062, China

**Keywords:** social media use, anxiety, adolescence, Chinese adolescents, mental health

## Abstract

The relationship between social media use and adolescent mental health is complex and multifaceted, with recent findings suggesting that the associations vary according to the nature of social media activities, but the most common social media usage patterns among Chinese adolescents remain insufficiently explored. This study explored the associations of overall social media use and social media use for entertainment and information seeking with anxiety among Chinese adolescents across three developmental stages. A total of 6125 adolescents (*M*_age_ = 14.65) from urban and suburban schools across four regions of China were included in the analyses. Participants completed surveys measuring the frequency, duration, and type of social media use, anxiety symptoms, and demographic characteristics. The results showed that, in the adjusted group analyses, entertainment-oriented social media engagement was significantly associated with higher anxiety, whereas information-seeking use showed no significant association with anxiety. Furthermore, age was identified as a significant moderator of the association between entertainment-oriented social media use and anxiety, with this association being particularly pronounced during early adolescence (ages 12–14). These findings may inform the development and evaluation of age-specific intervention strategies focused on entertainment-oriented social media engagement, particularly during the critical developmental window of early adolescence.

## 1. Introduction

Technological advancements have significantly altered interpersonal connections, with social media emerging as a dominant force in contemporary communication. Although these platforms have proliferated rapidly, reshaping how individuals share information and express themselves, their growing presence has also prompted concerns about emotional implications, especially during adolescence (Bell et al., 2015). Adolescence is marked by substantial psychological and biological development, making it a pivotal period in shaping long-term emotional health (Dahl et al., 2018). Given these developmental changes, exploring how social media engagement relates to emotional health during adolescence is necessary (Odgers & Jensen, 2020). However, existing studies have yielded inconsistent findings concerning social media use and adolescent mental health (Kerr et al., 2025; Odgers & Jensen, 2020). One possible reason is that assessments of overall use combine activities that differ in content, purpose, and social meaning. Examining these differences may help clarify why adolescents with similar levels of use do not necessarily report similar emotional experiences.

The Differential Susceptibility to Media Effects Model offers a framework for understanding why the association between social media use and emotional health may differ across adolescents. The model identifies dispositional characteristics, developmental stage, and social environment as factors that shape responses to media, while also emphasizing the cognitive and emotional experiences that arise during use (P. M. Valkenburg & Peter, 2013). Thus, the relevance of social media to mental health may depend on both what adolescents encounter online and how they interpret those experiences at a particular stage of development. Social Comparison Theory describes one process that can make online experiences personally meaningful: people evaluate aspects of themselves by comparing them with others (Festinger, 1954). On social media, selected portrayals of peers’ appearance, achievements, and social lives provide frequent opportunities for such comparison. Likes and comments can also make peer approval visible, inviting adolescents to consider how their own posts and experiences are received. In an adolescent study, technology-based social comparison and feedback seeking were associated with depressive symptoms after accounting for overall technology use and earlier symptoms (Nesi & Prinstein, 2015). An experimental social media task further showed that visible peer endorsement shaped adolescents’ responses to photographs (Sherman et al., 2016). Together, these findings suggest why engagement involving peer content, self-presentation, and feedback may carry particular emotional significance and why that significance could vary with adolescents’ developmental and social circumstances.

The experiences described above unfold within the platforms adolescents use and the social circumstances of their daily lives. In China, the most common activities on social media platforms among adolescents are learning and entertainment (e.g., viewing short videos and listening to music). It was reported that social media is frequently used by 54.1% of young users, primarily for entertainment (e.g., scrolling through short videos, posting selfies) and information gathering (e.g., staying updated on domestic and international news and major events) (China Internet Network Information Center, 2023). Recommendation feeds can bring peer-generated posts, entertainment, and informational content into the same stream. Research with Chinese adolescents has examined reliance on algorithmic recommendations alongside other short-video use behaviors, illustrating how platform design can shape patterns of engagement (Xu et al., 2023). These online experiences also take place alongside academic demands and family guidance. A longitudinal study of Chinese adolescents linked changes in academic pressure to later social anxiety through classmate support and problematic smartphone use (Zhao et al., 2024). Research with Chinese parents has documented active guidance, restrictions, and monitoring of adolescents’ social media use (L. Chen et al., 2023), while a study of Chinese children and adolescents examined social media use in relation to online learning and academic performance (Hou et al., 2024). Together, these findings place adolescents’ online activities within an environment shaped by recommendations, schooling, peer relationships, and parental guidance. This context may influence both the purposes for which adolescents use social media and the experiences they have while using it.

Among the psychological challenges related to social media usage, anxiety has emerged as one of the most critical concerns (Shannon et al., 2022). Adolescent-reported social media usage is related to anxiety (Woods & Scott, 2016). Prior research has indicated that prolonged social media usage could exacerbate anxiety symptoms and psychological distress, particularly among adolescents (Brailovskaia & Margraf, 2020; Islam et al., 2021). A recent systematic review further reported an association between social media use and anxiety among adolescents (Lopes et al., 2022). However, findings differ across studies. A longitudinal study, for example, found no association between social media usage and anxiety (Steinsbekk et al., 2023). Furthermore, existing literature has predominantly conceptualized social media use as a singular variable or categorized it into active and passive usage, leaving different purposes of use less clearly differentiated. Entertainment-oriented engagement deserves attention because it can include watching videos, viewing peers’ posts, sharing content, and responding to feedback. These activities may involve different experiences even when adolescents report using social media equally often. Viewing selected portrayals of others can invite comparison, while posting and reading responses can draw attention to peer feedback. Research on Chinese adolescents’ short-video use has also distinguished entertainment needs from social, self-presentation, and information needs, illustrating the value of examining purposes of engagement more closely (Zhu et al., 2024). These considerations provide a basis for examining whether the frequency of entertainment-oriented use is associated with anxiety across adolescence, alongside overall checking frequency and duration.

Alongside entertainment, adolescents also use social media to seek information. They may look for study materials, follow current events, or seek practical information through the same platforms they use for entertainment. These activities can offer useful knowledge, yet the emotional experience of seeking information may depend on its subject and presentation. Information concerning examinations, health, or uncertain events, for example, may be experienced differently from a practical explanation of a school topic. Research involving adolescents and young adults has identified entertainment and information seeking as distinct motives for social networking use (Pertegal et al., 2019). These purposes can nevertheless overlap in everyday use. A single short video may explain a school topic through humor or music, providing information and entertainment at the same time (Qin & Yin, 2026). Adolescents may also encounter entertaining posts while seeking information or move between these purposes within a single session. The differences between entertainment-oriented and information-seeking social media use may be associated with different psychological experiences, yet their respective associations with anxiety have not been sufficiently explored. Considering both purposes may help clarify how social media use relates to anxiety and whether these associations differ across adolescence.

Understanding how social media engagement patterns relate to mental health in early adolescence is important because adolescents’ online experiences may change as they develop. Indeed, social media may be particularly important at this stage, given that adolescents prioritize exploring identity and seeking peer approval (Gerwin et al., 2018). As adolescents grow, the developmental trajectory of their brains, particularly in systems involved in social cognition and emotional regulation, suggests heightened sensitivity to the benefits and detriments arising from social media interactions (Casey et al., 2019). Moreover, as adolescents navigate key transitions, such as physiological and environmental changes, they often encounter increased psychological and social pressures that may amplify their emotional reactions to online engagement (Crone & Konijn, 2018). Adolescence can typically be categorized into three stages: early, middle and late adolescence, based on age and educational level. Recent research has found that higher social media engagement in early adolescence was associated with increased odds of anxiety (Mundy et al., 2021). Other studies have documented that social media usage differentially affects the psychological health of early and late adolescents (Frison & Eggermont, 2015; Puukko et al., 2020; Salmela-Aro et al., 2017). Together, these findings support examining these stages separately when considering whether the association between social media use and anxiety differs across adolescence.

Considering the aforementioned findings, examining social media engagement patterns throughout adolescence, including early adolescence, may help clarify their associations with anxiety and may provide insights for targeted prevention measures aimed at promoting healthier online behaviors and supporting mental well-being across all stages of adolescence. In addition, inconsistencies in existing research findings may partly reflect differences in the regions and populations studied, while reliance on samples from specific locations may limit the generalizability of individual findings (Saleem et al., 2024). Therefore, it is important to include a broader range of sites in research on social media use and anxiety among Chinese adolescents.

The objective of the current study was therefore to explore the associations of overall social media use and specific patterns of engagement with anxiety levels among adolescents. Using data collected in four regions of China, we examined overall social media use and social media engagement for entertainment and information seeking and assessed whether their associations with anxiety differed across age groups. We hypothesized that there would be a positive association between social media use for entertainment and anxiety levels, with the association most pronounced in early adolescence.

## 2. Materials and Methods

### 2.1. Participants

**Figure 1:**
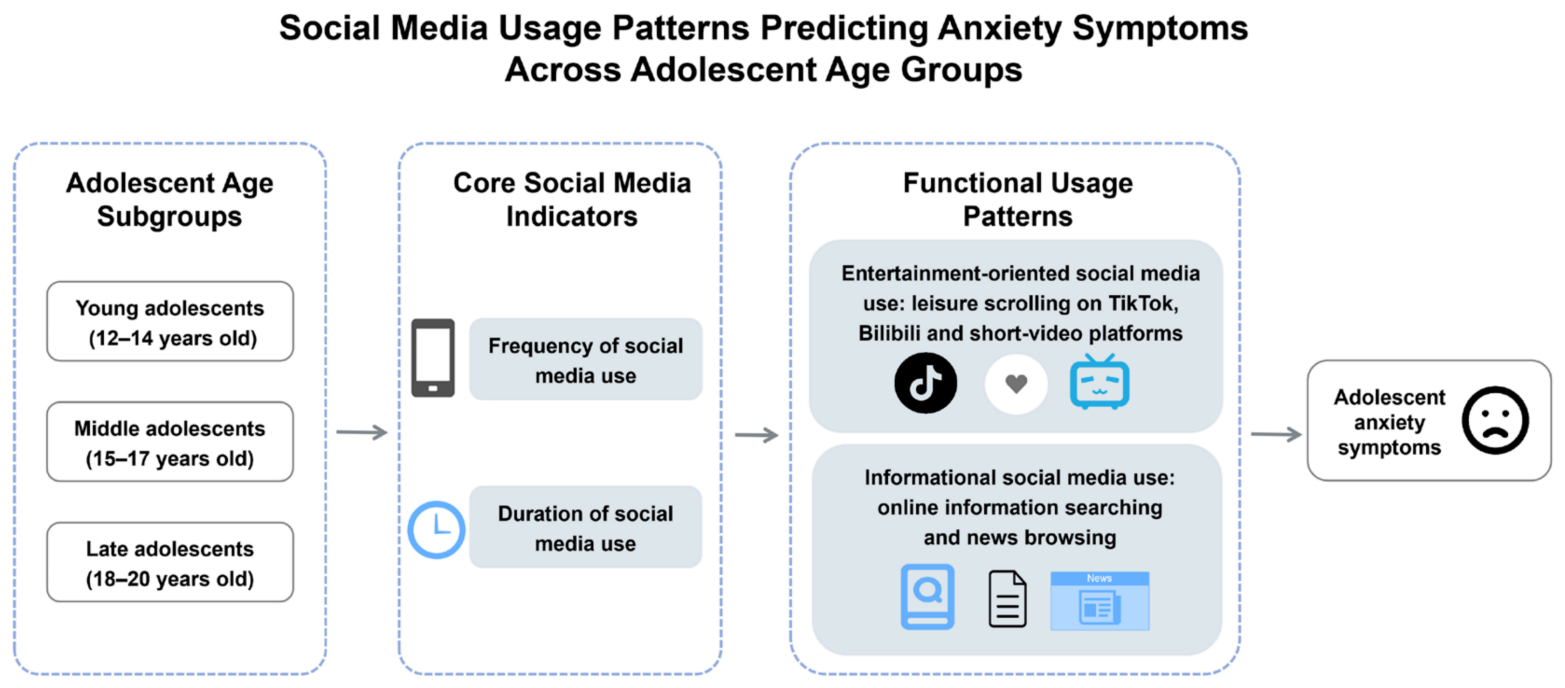
Social Media Usage Patterns Predicting Anxiety Symptoms Across Adolescent Age Groups.

The data for the participants were collected as part of a project on adolescent negative emotion assessment and intervention funded by the National Social Science Fund of China. The project aimed to collect comprehensive data on emotion, personality, and behavior from a large and diverse sample of Chinese youth, aged 10–20 years. The study recruited volunteers from four sites in China, including Chongqing, Shandong, Shanxi, and Gansu. Data for the current research were obtained from March 2023 to April 2024. Prior to data collection, permission was secured from the school administrations, and informed consent was obtained from the parents or guardians of minors. Minors also provided assent before completing the survey. All participants aged 18 or older signed written informed consent before completing the survey. Teachers presented the study during regular class hours. Students who wished to participate completed the survey during a subsequent supervised class session using the Wenjuanxing online survey platform. Three to five psychology graduate students served as research assistants and provided assistance when needed. Unique participant identifiers were assigned in advance, and recorded class and submission information was checked to prevent duplicate participation. All participants received a gift after data collection. Of the 6150 submitted questionnaires, 25 were excluded because of incomplete data. A total of 6125 participants (2944 females; mean age = 14.65 ± 2.06 years, age range: 12–20 years) were enrolled in the current study, consisting of 5603 middle or high school students and 522 university students. Participants were all native Chinese speakers. None of them reported a history of psychiatric or neurological disorders. By age, participants were classified as early adolescents (N = 2994, 1587 male, mean age = 12.87 ± 0.78 years, age range: 12–14 years), middle adolescents (N = 2609, 1354 male, mean age = 15.83 ± 0.70 years, age range: 15–17 years), or late adolescents (N = 522, 240 male, mean age = 18.90 ± 0.74 years, age range: 18–20 years), consistent with prior studies (Frison & Eggermont, 2015; Puukko et al., 2020; Salmela-Aro et al., 2017) (Figure 1). This study was conducted in accordance with the principles of the Declaration of Helsinki and its later amendments and was approved by the Southwest University Human Ethics Committee for Human Research (H23144).

### 2.2. Measures

#### 2.2.1. Demographic Variables

Participants reported their gender, age and study site. Household socioeconomic status (SES) was estimated from five questionnaire items: the presence of a separate study room, the numbers of bathrooms, air conditioners, and televisions or projectors in the home, and the number of books in the household. The responses to the five items were standardized, and principal component analysis was performed to derive a composite SES score.

#### 2.2.2. Use of Social Media

To assess overall social media usage, two items were adapted from Fardouly and Vartanian (2015). The frequency of checking social media (including WeChat, TikTok, Bilibili, etc.) was assessed on a 7-point scale, with options ranging from “never” to “once every two minutes or more frequently.” The duration of social media use was assessed on a 7-point scale, with intervals ranging from “less than 5 min” to “more than 6 h.” The scale has been used with Chinese adolescents and college students (Tie et al., 2024). We evaluated participants’ use of social media for two specific activities: entertainment and information gathering. Entertainment and information gathering have been examined as distinct reasons for social media use (Pertegal et al., 2019). For each activity, participants indicated whether they used social media for entertainment (e.g., watching videos or posting selfies) and for searching for information (e.g., looking up study materials or other information) (yes/no for each activity) and reported how often they used social media for that activity using one frequency item, rated on a 5-point scale: “Never,” “Rarely,” “Sometimes,” “Often,” and “Always.” These response options were aligned with those used in previous research (Gerson et al., 2017). The two frequency items were scored separately, and participants could report frequent use for both activities. Each item therefore represents the self-reported frequency of the specified activity rather than a comprehensive measure of social media use motivations.

#### 2.2.3. Anxiety Level

The Generalized Anxiety Disorder 7-item (GAD-7) scale was used to assess the anxiety symptoms of the participants. Participants were asked to rate the degree to which they had been bothered by each of the seven items over the past two weeks. The items are rated on a 4-point Likert scale, with responses from 0 (“not at all”) to 3 (“nearly every day”). The scores were summed to yield a total score ranging from 0 to 21, with higher scores indicating more frequent anxiety symptoms over the past two weeks. The total score was used as a continuous measure of anxiety symptom severity, rather than as a clinical diagnosis. The questionnaire was originally validated as a screening instrument (Spitzer et al., 2006), and subsequent studies have supported the reliability and validity of its total score in large samples of Chinese adolescents (Sun et al., 2021) and adolescents in general-population and educational settings (Casares et al., 2024; Tiirikainen et al., 2019). For descriptive interpretation, we used the conventional GAD-7 symptom severity cut points of 5, 10, and 15 (Spitzer et al., 2006). The distribution of GAD-7 scores across conventional symptom severity ranges and cut points is presented in Supplementary Table S1.

### 2.3. Data Analysis

Statistical analyses were implemented in SPSS software (version 25.0; IBM, Armonk, NY, USA) and jamovi (version 2.6.26.0). Descriptive data were presented as mean ± SD, and the GAD-7 total score was additionally summarized using the median and interquartile range (IQR; Supplementary Table S1). Pearson partial correlations were used to assess associations among the study variables in the full analytic sample, controlling for gender, age, SES, and study site. To compare participants at the lower and upper ends of each response scale, checking frequency and daily duration were classified as low (scores 1–2) or high (scores 6–7), and entertainment and information-seeking frequency were classified as low (scores 1–2) or high (scores 4–5). Participants with intermediate scores were excluded from the corresponding extreme-group comparison but remained in the full-sample correlational analyses. Age group by usage group comparisons were examined using two-way analyses of variance (ANOVAs), with homogeneity of variance assessed using Levene’s test, followed by Bonferroni post hoc tests. Analyses of covariance (ANCOVAs) additionally adjusted for gender, SES, and study site. To assess whether the findings depended on the extreme-group classification, we conducted separate full-sample ANCOVAs for each social media use item, retaining all original response categories and testing the interaction between age group and response category. These analyses also adjusted for gender, SES, and study site. The low- and high-use categories were selected symmetrically from the ends of each ordered response scale to provide focused comparisons between markedly different levels of use; they were not intended as validated risk thresholds. Residual normality was examined using Shapiro–Wilk tests, and homogeneity of variance was assessed using Levene’s test. Given departures from these assumptions, the four extreme-group ANCOVAs were repeated using HC3 heteroscedasticity-consistent standard errors, with the same factors, covariates, and interaction terms as in the primary analyses. Effect sizes were expressed as partial eta squared (*η*^2^*_p_*), and statistical significance was set at *p* < 0.05.

## 3. Results

### 3.1. Demographic Information

**Figure 2:**
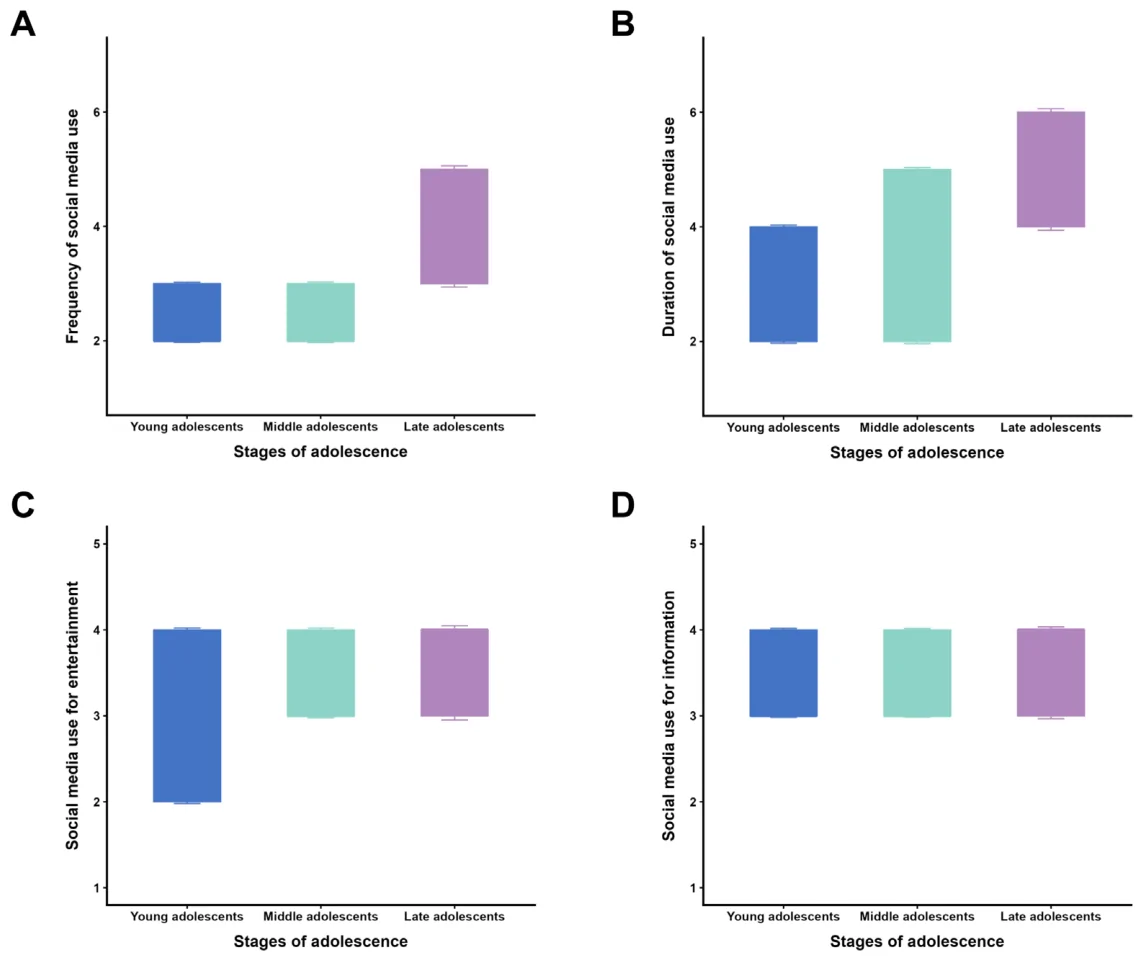
Social media usage across three adolescent age groups. Frequency of social media use (**A**), duration of social media use (**B**), social media use for entertainment (**C**) and social media use for information (**D**) across three adolescent age groups.

The social media usage across adolescent age groups is presented in Figure 2. Adolescents aged 12–14 and 15–17 reported mean frequency of social media use scores of 2.72 ± 1.46 and 2.83 ± 1.55, respectively (Table 1). Many youths in these groups may have limited autonomy in accessing social media, as parental restrictions on mobile device usage and social media account registration are generally stricter during early adolescence, in contrast to university years.

### 3.2. Associations Between Social Media Use and Anxiety

We first examined the associations between social media use and anxiety level using Pearson partial correlations, controlling for age, sex, SES, and study site. As predicted, there were significant positive correlations between the anxiety level and overall social media use (frequency: *r* = 0.18, *p* < 0.001; duration: *r* = 0.16, *p* < 0.001), social media use for entertainment (*r* = 0.13, *p* < 0.001), and social media use for information (*r* = 0.08, *p* < 0.001) (Table 2). These results indicate that greater self-reported social media engagement was associated with higher anxiety levels, regardless of whether social media was used for entertainment or information.

### 3.3. The Effect of Overall Social Media Use on Anxiety

**Figure 3:**
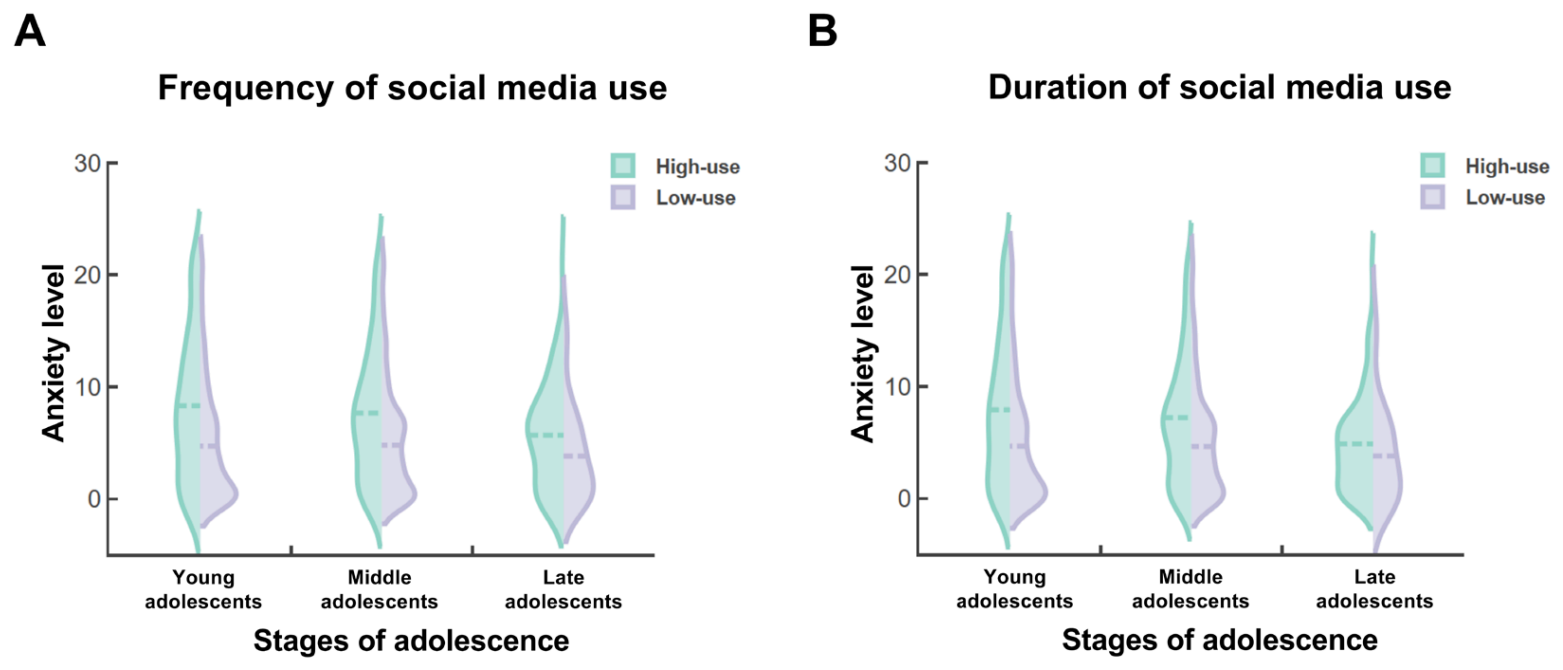
Results of overall social media use on anxiety. Effects of frequency of social media use (**A**) and duration of social media use (**B**) on anxiety levels across three adolescent age groups.

We examined the associations of overall social media use with anxiety across age groups using separate ANCOVAs for checking frequency and daily duration, adjusting for gender, SES, and study site. Significant main effects of overall social media use were found (frequency: *F*_(1,3757)_ = 66.39, *p* < 0.001, *η*^2^*_p_* = 0.02; duration: *F*_(1,2973)_ = 36.08, *p* < 0.001, *η*^2^*_p_* = 0.01), with higher anxiety levels in the high-use groups overall. Age group also showed significant main effects in the checking-frequency model, *F*_(2,3757)_ = 7.34, *p* < 0.001, *η*^2^*_p_* = 0.01, and the duration model, *F*_(2,2973)_ = 6.86, *p* = 0.001, *η*^2^*_p_* = 0.01, revealing significant differences in anxiety values among the three age groups. Post hoc tests further revealed that late adolescents exhibited significantly lower anxiety levels than both early and middle adolescents. Bonferroni-adjusted pairwise comparisons showed that late adolescents had lower anxiety levels than early adolescents in both the frequency model (mean difference = 1.85, *p* < 0.001) and the duration model (mean difference = 1.99, *p* < 0.001). Late adolescents also had lower anxiety levels than middle adolescents in both models (frequency: mean difference = 1.55, *p* = 0.004; duration: mean difference = 1.62, *p* = 0.009). Early and middle adolescents did not differ significantly (frequency: mean difference = 0.30, *p* = 0.718; duration: mean difference = 0.37, *p* = 0.316). The interactions between age groups and social media use groups were not significant (frequency: *F*_(2,3757)_ = 2.26, *p* = 0.105; duration: *F*_(2,2973)_ = 2.59, *p* = 0.075) (Figure 3). The analyses retaining all original response categories likewise showed no significant age group × response category interaction for either measure (Supplementary Table S2).

### 3.4. The Effect of Social Media Usage Patterns on Anxiety

**Figure 4:**
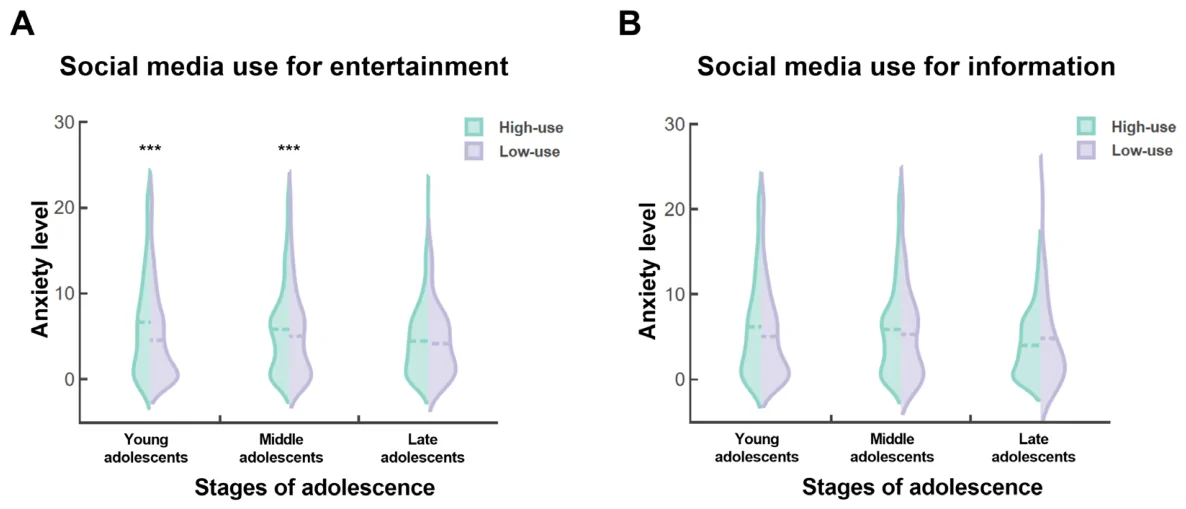
Results of different social media use patterns on anxiety. Effects of social media use for entertainment (**A**) and social media use for information (**B**) on anxiety levels across three adolescent age groups. *** *p* < 0.001 vs. late-adolescent group.

To explore the associations of social media use for entertainment and information with anxiety, we analyzed the frequency of both social media usage patterns using separate ANCOVAs, adjusting for gender, SES, and study site. For the social media engagement for entertainment, a significant main effect was found for use group (*F*_(1,3541)_ = 12.73, *p* < 0.001, *η*^2^*_p_* = 0.01). Additionally, a significant main effect of age group was observed (*F*_(2,3541)_ = 8.91, *p* < 0.001, *η*^2^*_p_* = 0.01). Bonferroni-adjusted pairwise comparisons showed that early and middle adolescents had higher anxiety levels than late adolescents (mean differences = 1.63 and 1.54, respectively; both *p* < 0.001), whereas early and middle adolescents did not differ significantly (mean difference = 0.09, *p* = 1.000). The interaction between age groups and social media use for entertainment was significant, *F*_(2,3541)_ = 8.07, *p* < 0.001, *η*^2^*_p_* = 0.01 (Figure 4A), indicating that age moderated the association between anxiety and entertainment-oriented social media engagement, with the difference between high- and low-use groups decreasing from early to late adolescence. Within early adolescence, the high-use group had higher anxiety levels than the low-use group (mean difference = 2.08, Bonferroni-adjusted *p* < 0.001). The corresponding differences were not significant in middle adolescence (mean difference = 0.67, *p* = 0.370) or late adolescence (mean difference = 0.16, *p* = 1.000). However, for the information-seeking use, neither the main effect of use group, *F*_(1,3034)_ = 0.47, *p* = 0.492, nor that of age group, *F*_(2,3034)_ = 2.02, *p* = 0.133, was significant. The interaction between age groups and information-seeking social media engagement was not significant (*F*_(2,3034)_ = 1.90, *p* = 0.149) (Figure 4B). In the analyses retaining all original response categories, the age group × response category interaction remained significant for entertainment use but was not significant for information-seeking use (Supplementary Table S2). HC3 sensitivity analyses did not change the pattern of statistical conclusions for the four primary ANCOVAs (see Supplementary Results).

### 3.5. The Moderating Effect of Age on the Association Between Social Media Use for Entertainment and Anxiety Across Genders

**Figure 5:**
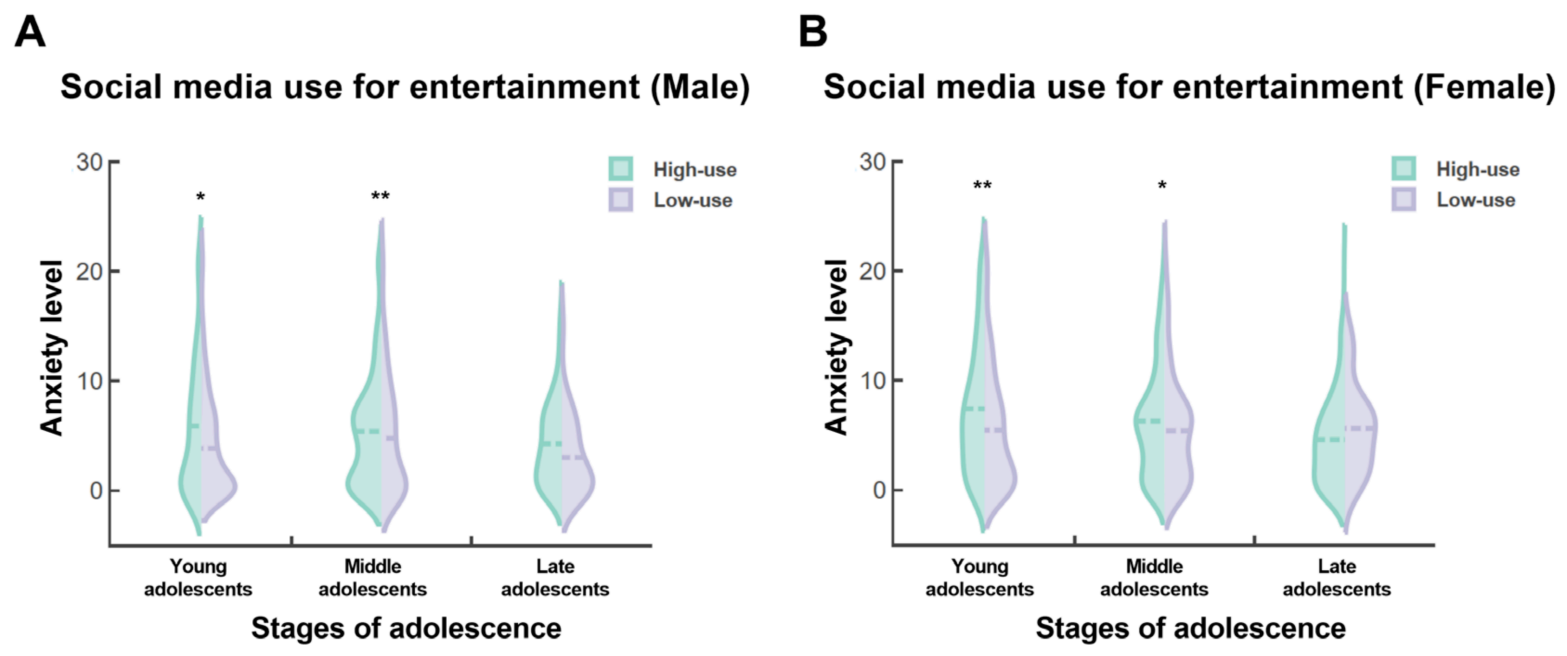
Results of social media use for entertainment on anxiety across genders. Effects of social media use for entertainment on anxiety levels in male (**A**) and female (**B**) adolescents across three age groups. * *p* < 0.05, ** *p* < 0.01 vs. late-adolescent group.

To examine the stability of the moderating effect of age across genders, we analyzed the data separately for males and females using ANCOVAs adjusted for SES and study site. The results showed that both the main effects of social media engagement for entertainment and age group were significant, and the interaction between age groups and social media engagement for entertainment was also significant for both males (*F*_(2,1867)_ = 3.83, *p* = 0.022, *η*^2^*_p_* = 0.01) and females (*F*_(2,1665)_ = 4.85, *p* = 0.008, *η*^2^*_p_* = 0.01) (Figure 5), indicating that age moderated the association between entertainment-oriented social media engagement and anxiety across gender. Bonferroni-adjusted pairwise comparisons showed that, among early adolescents, the high-use group had higher anxiety levels than the low-use group in both males (mean difference = 2.03, *p* < 0.001) and females (mean difference = 2.08, *p* < 0.001). The corresponding differences were not significant in middle or late adolescence for either gender. Thus, the association between high entertainment use and higher anxiety during early adolescence was observed in both the male and female analyses. These findings suggest that age group is an important consideration when examining the association between social media engagement for entertainment and anxiety, regardless of gender.

## 4. Discussion

The present study investigated the association between social media use and anxiety in adolescence by examining different types of social media engagement across three developmental stages. The results identified positive associations between self-reported social media use and anxiety symptoms, suggesting that increased engagement with these platforms may be associated with higher anxiety levels. This finding is consistent with previous research by Keles et al. (2020), which reported that higher social media engagement is associated with elevated anxiety in adolescents. Similarly, Weigle and Shafi (2024) described a link between social media engagement and increased anxiety and depressive symptoms. Prior findings have also differed across studies. Some have reported associations with poorer mental health, whereas others have found weak or non-significant associations (Kerr et al., 2025; Steinsbekk et al., 2023). Such differences may reflect variation in the activities adolescents undertake online, their developmental stage, and their individual experiences. For example, Orben et al. (2022) reported age-related differences in the association between social media engagement and life satisfaction, while an experience-sampling study found considerable variation between adolescents in the relationship between social media use and self-esteem (P. Valkenburg et al., 2021). Anxiety may also influence subsequent use. A longitudinal study of Chinese adolescents found bidirectional associations between anxiety symptoms and problematic short-form video use, although that construct differs from the activity frequency measured here (Li et al., 2025).

These conflicting findings suggest that differences in adolescents’ online experiences warrant closer examination. To explore this further, we examined how entertainment-oriented and information-seeking social media engagement are associated with anxiety. Our analysis revealed distinct patterns, showing that the associations with anxiety vary based on whether the purpose is entertainment or information. In the adjusted group analyses, entertainment-oriented social media engagement showed a statistically significant main effect and an interaction with age group, whereas the corresponding main effect and interaction for information-seeking engagement were not significant. The age-group interaction for entertainment use was also observed when all original response categories were retained. Together, these results suggest that broad descriptions of time spent on social media may conceal differences in the activities and experiences associated with use. These results are consistent with prior research indicating that social media engagement for entertainment, such as browsing for leisure, viewing peers’ posts, posting content, reading comments, and interacting with influencer content, was linked to negative emotional outcomes (Appel et al., 2016). In contrast, information-seeking behaviors, such as browsing educational content or news, might not invoke the same level of emotional engagement and, therefore, may differ in their associations with anxiety. These activities can also occur during the same session. A short video may also be informative and entertaining at the same time (Qin & Yin, 2026). Their relevance to anxiety may therefore depend on what adolescents encounter and how they engage with it, in addition to how frequently they use a platform.

Social comparison offers one possible explanation for the association involving entertainment use. Users may present selected, positive facets of their lives on their social media profiles. This selective portrayal can create a distorted sense of reality, particularly when social media is used primarily for entertainment purposes. In these contexts, exposure to curated content may foster social comparisons even during passive viewing. Recommendation feeds may repeatedly expose adolescents who only browse to socially salient content, even when they do not post or comment (Winstone et al., 2026). Images and visible popularity indicators can make appearance, achievement, and peer approval salient during browsing (Nesi et al., 2018). Such comparisons may be accompanied by unfavorable self-evaluations and negative emotions (Appel et al., 2016). In a study of adolescents, technology-based social comparison and feedback-seeking were associated with depressive symptoms even after accounting for overall technology use and earlier symptoms (Nesi & Prinstein, 2015). Recent research reported greater body image concerns among social media users than non-users (Fardouly et al., 2020; Tie et al., 2024). Longitudinal research further found that appearance comparisons, including those made while using social media, predicted later social anxiety symptoms (Rapee et al., 2022). Entertainment feeds that emphasize appearance, achievement, or peer approval could provide repeated opportunities for unfavorable self-evaluation, particularly for adolescents who attach importance to those domains.

Online self-presentation during social media use for entertainment may also be relevant to psychological well-being. A heightened focus on self-presentation on social media has been related to increased anxiety and reduced well-being (Hawes et al., 2020). Nesi et al. (2018) described how public comments and other feedback features can make peer responses visible and may shape adolescents’ excessive interpersonal feedback-seeking and self-evaluations, resulting in negative self-assessments and emotions. In experimental research, adolescents who received fewer likes reported stronger feelings of rejection and more negative affect (Lee et al., 2020). In an experimental social media task, adolescents were more likely to endorse photographs that had received many likes, showing that visible peer approval can shape responses to online content (Sherman et al., 2016). Concerns about peer acceptance may make such feedback particularly meaningful during a developmental period in which peer relationships and identity are changing (Gerwin et al., 2018; Spies Shapiro & Margolin, 2014). Posting content and monitoring responses involve direct feedback about oneself, whereas adolescents who only view content can still see how others are received. This account need not apply equally to every form of entertainment use. Watching a video, comparing oneself with its creator, posting a selfie, and receiving comments involve different social experiences. Distinguishing passive viewing from active posting and feedback-seeking would help identify which experiences are most closely associated with anxiety.

FoMO and the demands of continuous connection provide further possible explanations. Adolescents may check social media to remain aware of peer activities or respond to concerns that they are missing social events (Beyens et al., 2016). In a separate adolescent sample, FoMO moderated the association between frequency of social media use and anxiety symptoms, with more frequent use associated with higher anxiety among adolescents reporting greater FoMO (Einstein et al., 2023). FoMO may accompany passive monitoring of peers’ activities, while the availability of notifications and expectations of a prompt response can make frequent checking part of active social exchange (Beyens et al., 2016; Nesi et al., 2018). Other experiences discussed in prior research include feeling that time online has been wasted (Sagioglou & Greitemeyer, 2014), communication overload (W. Chen & Lee, 2013), and negative social interactions (Glover & Fritsch, 2018). Research on digital media use and mobile phones at bedtime has also reported that regular checking of social media can contribute to anxiety related to FoMO, offering another reason to examine when frequent checking occurs (LeBourgeois et al., 2017; Vernon et al., 2018). These processes may occur during either entertainment or information-seeking use, since information seeking can also involve emotionally salient news or social feedback. Social comparison, FoMO, and feedback-seeking thus provide literature-supported possibilities for interpreting our findings. Taken together, these findings suggest that the purpose and experiences of social media use may matter alongside overall time spent online when examining anxiety.

Although some research on individual differences has focused on college populations (Correa et al., 2010), early adolescence may warrant attention because online experiences occur alongside rapid developmental change. Our findings showed a significant main effect of age, with late adolescents reporting lower anxiety levels than early and middle adolescents in the checking-frequency, duration, and entertainment-use models. Moreover, the association between social media engagement for entertainment and anxiety differed across age groups. The difference between the high- and low-entertainment-use groups was significant in early adolescence and was smaller in the older groups, consistent with the interaction between the age group and entertainment-use group. This pattern is notable because early adolescents did not report the highest average frequency of overall social media use (Table 1). Developmental context may therefore be relevant alongside the amount of use. Orben et al. (2022) also reported that the association between life satisfaction and social media use varied across adolescence, with early adolescence being a critical period. During the ages of 12–14, adolescents experience significant developmental changes, both physically and psychologically. This period is marked by heightened sensitivity to peer influence, rapid changes in brain development, and the ongoing formation of self-identity (Gerwin et al., 2018; Vankerckhoven et al., 2023). Social media may be particularly salient during this stage because it offers frequent opportunities for social comparison, which may be associated with anxiety and insecurity. Moreover, the heightened need for social approval during this stage could exacerbate social comparison, intensifying feelings of insecurity, particularly during a period when identity formation is still developing (Vankerckhoven et al., 2023). Longitudinal evidence that appearance comparisons predicted later social anxiety during this period (Rapee et al., 2022), together with findings on responses to visible peer endorsement (Sherman et al., 2016), suggests reasons to examine these experiences more closely in early adolescence. At the same time, age groups may also differ in schooling, autonomy, and academic pressures, which future longitudinal studies could examine alongside online experiences.

Our findings suggest that the association between estimated social media use for entertainment and anxiety differs across age groups, with similar patterns observed in the separate analyses of males and females. Within early adolescence, the high-use group reported higher anxiety than the low-use group among both males and females. In another study on social media use among high school students, Frison and Eggermont (2016) reported a gender difference in passive Facebook usage and depression. While gender differences in social media usage and psychological well-being have been noted (Orben et al., 2022), our findings suggest that the developmental trajectory of anxiety in response to entertainment use may be more universal. Because the gender composition of the sample differed across age groups, adjusting for gender in the primary models and examining males and females separately provides additional context for interpreting this pattern. A possible explanation for the age-related pattern lies in the developmental characteristics of adolescence. During this critical period of identity and self-concept development, adolescents may be sensitive to social comparison and online self-presentation during entertainment engagement (Vankerckhoven et al., 2023). These similar patterns across genders suggest that the mechanisms underlying the association between entertainment-oriented engagement and anxiety may be associated with broader psychosocial processes. These findings highlight the value of considering developmental stage alongside gender when examining the association between social media usage and anxiety.

These findings add to research suggesting that the associations between social media use and mental health may be better understood by considering what adolescents do online and when in development they do it, alongside overall frequency or duration. Entertainment and information seeking can overlap within the same session and need not define exclusive groups of users. Passive viewing and active interaction may also involve different experiences within either purpose of use. Future studies could distinguish viewing from posting, identify the content encountered, and measure social comparison, feedback-seeking, and FoMO directly. Repeated assessments would help examine whether an adolescent’s anxiety changes following particular online experiences and whether existing anxiety predicts subsequent patterns of use. This approach may clarify why average associations can be modest even when online experiences differ substantially across individuals (P. Valkenburg et al., 2021).

These findings also have implications for support at school and at home. For younger adolescents, guidance could address the experiences associated with entertainment use alongside frequency and total screen time. Schools could incorporate social media literacy into existing health education through discussion of selective self-presentation, social comparison, and responses to online feedback. Students could practice distinguishing supportive interactions from unfavorable comparisons and recognizing when online activity leaves them distressed or excluded. Students could also learn how recommendations shape what appears in a feed and discuss ways to seek support after uncomfortable online experiences. A school-based social media literacy trial provided preliminary support for some body-image-related outcomes (Gordon et al., 2021). Families could discuss the content adolescents encounter, invite them to describe uncomfortable comparisons or peer feedback, and agree on routines for breaks and offline activities. Longitudinal work with early adolescents linked active media parenting with later mental health through social efficacy, while restrictive parenting was associated with reduced online risk-taking rather than later mental health in that sample (Doty et al., 2025). Parents and adolescents could also review notification and recommendation settings together and revisit their agreements as adolescents gain greater independence. Brief breaks from social media have also been studied in relation to well-being and perceived stress (Fioravanti et al., 2020; Turel et al., 2018), and their relevance to younger adolescents could be evaluated. Approaches that encourage meaningful online interaction and developmentally appropriate boundaries may be worth examining alongside simple limits on use.

Platform design deserves attention as well. Interviews with adolescents have described how algorithmic recommendations can repeatedly present emotionally salient content, while adolescents’ attempts to manage their feeds may be limited by the available controls (Winstone et al., 2026). Platforms could offer clearer controls over recommendations and notifications, easier ways to reduce repeated exposure to unwanted content, and age-appropriate explanations of why content is being recommended. Platforms could also give younger users more choice over the visibility of engagement counts and the public feedback attached to their posts. Given the salience of visible peer endorsement (Sherman et al., 2016), options for managing exposure to popularity metrics and public feedback may also merit study. Such features could be developed with adolescents and evaluated for their effects on online experiences and mental health.

### Limitations

This study is subject to several limitations. First, the current cross-sectional study does not establish the direction of the association between social media use and anxiety. Adolescents with higher anxiety symptoms may also be more likely to use social media for entertainment. The observed associations were modest, and factors not measured here, such as academic pressure and prior mental health, may contribute to them. Future longitudinal studies that assess these factors could clarify how social media use and anxiety relate over time. Second, although participants were recruited from middle schools, high schools, and universities at four study sites, the sample was not nationally representative. Therefore, caution is warranted when generalizing these findings to adolescents in other parts of China, including highly developed eastern coastal cities, or to other cultural contexts. Finally, entertainment and information-seeking use were assessed with single self-report items, for which internal consistency could not be evaluated. Self-reported use may also differ from objectively recorded activity. The items did not distinguish specific platforms or content, passive viewing from active interaction, or situations in which entertainment and information seeking overlap. Moreover, the social comparison, FoMO, and peer feedback processes discussed above were not measured. Future studies could combine more detailed measures of online activities and these potential mechanisms with device-recorded usage logs.

## 5. Conclusions

In conclusion, our findings indicate that the association between social media use and anxiety in adolescence varies across forms of engagement and developmental stages. This study highlights entertainment-oriented social media use as particularly relevant to anxiety, whereas information-seeking use did not show the same age-related pattern. Furthermore, the association between entertainment-oriented use and anxiety was moderated by age, with the difference between high- and low-use groups most pronounced in early adolescence. This pattern suggests that early adolescence may be a particularly important developmental period for understanding this association. Inconsistencies in previous research may partly reflect the limited differentiation of social media usage patterns. This research contributes to understanding how the association between social media use and anxiety varies across adolescence and may inform the development and evaluation of age-appropriate guidance that considers adolescents’ specific social media engagement.

## Figures and Tables

**Table 1:** Descriptive statistics of the demographic data.

Demographic	Young Adolescents Aged 12–14 (n = 2994)	Middle Adolescents Aged 15–17 (n = 2609)	Late Adolescents Aged 18–20 (n = 522)
Gender (F/M)	1407/1587	1255/1354	282/240
Mean age	12.87 ± 0.78	15.83 ± 0.70	18.90 ± 0.74
Frequency of social media use	2.72 ± 1.46	2.83 ± 1.55	3.90 ± 1.35
Duration of social media use	3.14 ± 1.68	3.53 ± 1.81	5.04 ± 1.39
Social media use for entertainment	2.77 ± 1.18	3.27 ± 1.08	3.48 ± 1.08
Social media use for information	3.02 ± 0.98	3.41 ± 0.84	3.70 ± 0.78
Anxiety level	5.36 ± 5.37	5.33 ± 4.95	4.10 ± 4.01

**Table 2:** Means and standard deviations of variable values and correlations between the variables.

	1	2	3	4	5
1. Frequency of social media use	1				
2. Duration of social media use	0.61 ***	1			
3. Social media use for entertainment	0.33 ***	0.40 ***	1		
4. Social media use for information	0.24 ***	0.29 ***	0.43 ***	1	
5. Anxiety level	0.18 ***	0.16 ***	0.13 ***	0.08 ***	1
M	2.86	3.47	3.05	3.24	5.24
SD	1.52	1.79	1.16	0.94	5.10

Note: *** *p* < 0.001.

## Data Availability

The data presented in this study are available on request from the corresponding author due to privacy considerations concerning individual-level data collected from adolescent participants.

## Supplementary Materials

The following supporting information can be downloaded at: https://www.mdpi.com/article/10.3390/bs16101895/s1, Table S1. Distribution of GAD-7 scores across conventional anxiety symptom severity ranges and cut points in the analytic sample (N = 6125). Table S2. Full-sample ANCOVAs examining age group, original social media use response categories, and their interactions in relation to GAD-7 scores (N = 6125).
