# Supplementary material for: Social Media Usage Patterns and Anxiety in Chinese Adolescents: Age-Specific Risks from a Cross-Sectional Perspective
Source: Behav Sci (Basel). 2026 Oct 14;16(10):1895. doi: 10.3390/bs16101895 (PMC13701303; doi:10.3390/bs16101895)
Supplement: Supplementary file 1 [file behavsci-16-01895-s001.zip › behavsci-4600749-supplementary.pdf]

Supplementary Table S1. Distribution of GAD-7 scores across conventional anxiety symptom severity ranges and cut points in the analytic sample (N = 6,125)

GAD-7 score interpretation	Total score	Participants, n	Participants, %
Minimal	0–4	3,155	51.51
Mild	5–9	1,871	30.55
Moderate	10–14	737	12.03
Severe	15–21	362	5.91

Note. The median GAD-7 total score was 4 (IQR: 1-8).

Supplementary Table S2. Full-sample ANCOVAs examining age group, original social media use response categories, and their interactions in relation to GAD-7 scores (N = 6,125).

Social media use	Effect	df	*F*	*p*
Frequency	Age group	2, 6099	8.36	< .001
	Response	6, 6099	13.24	< .001
	Age group × Response	12, 6099	1.03	0.416
Duration	Age group	2, 6099	21.38	< .001
	Response	6, 6099	20.87	< .001
	Age group × Response	12, 6099	1.50	0.115
Entertainment	Age group	2, 6105	14.45	< .001
	Response	4, 6105	18.18	< .001
	Age group × Response	8, 6105	5.13	< .001
Information	Age group	2, 6105	1.68	0.186
	Response	4, 6105	0.71	0.587
	Age group × Response	8, 6105	0.79	0.611

Note. All original response categories were retained: seven each for checking frequency and daily duration, and five each for entertainment and information-seeking frequency. Models adjusted for gender, SES, and study sites.

Supplementary Results: Model diagnostics and HC3 sensitivity analyses

For the four extreme-group ANCOVAs, residual Shapiro-Wilk tests indicated departures from normality, and Levene's tests indicated unequal variances (Levene's test p values < .001). We therefore repeated these models using HC3 heteroscedasticity-consistent standard errors, retaining the same factors, covariates, interaction terms, and analytic samples as in the primary analyses.

The HC3-adjusted tests yielded the same pattern of statistical significance for the age-group and social media use main effects and their interactions as the primary ANCOVAs. The age group × checking frequency interaction was non-significant, $F_{(2, 3757)} = 2.17$, $p = 0.115$, as were the age group × duration interaction, $F_{(2, 2973)} = 2.76$, $p = 0.063$, and the age group × information-seeking use interaction, $F_{(2, 3034)} = 2.63$, $p = 0.072$. The age group × entertainment use interaction remained significant, $F_{(2, 3541)} = 8.66$, $p < 0.001$.
